# Gene targets for engineering osmotolerance in *Caldicellulosiruptor bescii*

**DOI:** 10.1186/s13068-020-01690-3

**Published:** 2020-03-13

**Authors:** Kyle B. Sander, Daehwan Chung, Dawn M. Klingeman, Richard J. Giannone, Miguel Rodriguez, Jason Whitham, Robert L. Hettich, Brian H. Davison, Janet Westpheling, Steven D. Brown

**Affiliations:** 1 BioEnergy Science Center, Oak Ridge National Laboratory, Oak Ridge, TN USA; 2 Bredesen Center for Interdisciplinary Graduate Research and Education, University of Tennessee, Knoxville, TN USA; 3 Department of Chemical and Biomolecular Engineering, University of Tennessee, Knoxville, TN USA; 4 Department of Genetics, University of Georgia, Athens, GA USA; 5 Biosciences Division, Oak Ridge National Laboratory, Oak Ridge, TN USA; 6 Chemical Sciences Division, Oak Ridge National Laboratory, Oak Ridge, TN 37830 USA; 7 Present Address: Department of Bioengineering, University of California, Berkeley, Berkeley, CA USA; 8 Present Address: National Bioenergy Center, National Renewable Energy Laboratory, Golden, CO USA; 9 Present Address: LanzaTech, Skokie, IL USA; 10 Present Address: Becton Dickinson Diagnostics, Sparks Glencoe, MD USA

**Keywords:** *Caldicellulosiruptor bescii*, Osmotolerance, Fatty acid biosynthesis, *dnaK*, *fapR*, *fruR/cra*

## Abstract

**Background:**

*Caldicellulosiruptor bescii*, a promising biocatalyst being developed for use in consolidated bioprocessing of lignocellulosic materials to ethanol, grows poorly and has reduced conversion at elevated medium osmolarities. Increasing tolerance to elevated fermentation osmolarities is desired to enable performance necessary of a consolidated bioprocessing (CBP) biocatalyst.

**Results:**

Two strains of *C. bescii* showing growth phenotypes in elevated osmolarity conditions were identified. The first strain, ORCB001, carried a deletion of the FapR fatty acid biosynthesis and malonyl-CoA metabolism repressor and had a severe growth defect when grown in high-osmolarity conditions—introduced as the addition of either ethanol, NaCl, glycerol, or glucose to growth media. The second strain, ORCB002, displayed a growth rate over three times higher than its genetic parent when grown in high-osmolarity medium. Unexpectedly, a genetic complement ORCB002 exhibited improved growth, failing to revert the observed phenotype, and suggesting that mutations other than the deleted transcription factor (the *fruR/cra* gene) are responsible for the growth phenotype observed in ORCB002. Genome resequencing identified several other genomic alterations (three deleted regions, three substitution mutations, one silent mutation, and one frameshift mutation), which may be responsible for the observed increase in osmolarity tolerance in the *fruR*/*cra*-deficient strain, including a substitution mutation in *dnaK*, a gene previously implicated in osmoresistance in bacteria. Differential expression analysis and transcription factor binding site inference indicates that FapR negatively regulates malonyl-CoA and fatty acid biosynthesis, as it does in many other bacteria. FruR/Cra regulates neighboring fructose metabolism genes, as well as other genes in global manner.

**Conclusions:**

Two systems able to effect tolerance to elevated osmolarities in *C. bescii* are identified. The first is fatty acid biosynthesis. The other is likely the result of one or more unintended, secondary mutations present in another transcription factor deletion strain. Though the locus/loci and mechanism(s) responsible remain unknown, candidate mutations are identified, including a mutation in the *dnaK* chaperone coding sequence. These results illustrate both the promise of targeted regulatory manipulation for osmotolerance (in the case of *fapR*) and the challenges (in the case of *fruR/cra*).

## Background

Consolidated bioprocessing (CBP) is projected to be among the least expensive processes being developed for the synthesis of ethanol from biomass [31]. Critical to an effective and economically viable CBP process is a biocatalyst organism capable of yield, productivity, and final titer metrics required by such a process. Currently, no such organism exists and active efforts to engineer such an organism are underway. Each biocatalyst organism being engineered suffers from its own inherent limitations [3, 24, 28, 58]. *Caldicellulosiruptor bescii*, a promising lignocellulolytic hyperthermophilic candidate CBP biocatalyst, is sensitive to elevated osmolarities, showing pronounced growth defects at a fraction of the projected final osmolarities of CBP fermentations [3, 9, 15]. A large increase in the effective osmotolerance in *C. bescii* is a prerequisite to be used as an industrial CBP biocatalyst.

Osmolarity stress in bacteria, in the context of bioprocessing, is typically brought about by elevated osmolarity in the extracellular environment exceeding that of the intracellular environment, causing intracellular influx of solutes and efflux of water. This decreases water activity inside of the cell and increases the intracellular concentration of potentially inhibitory solutes. Changes in growth environment osmolarity has also been shown to affect cellular attachment [54], which metabolically interacts with other cellular stress response systems, through pleiotropic regulators, such as carbon catabolite repression [1] and the expression of sigma factors [21].

Resistance to elevated osmolarity in bacteria is enabled through many single or combined physiological responses. Some bacteria are known to actively regulate their intracellular ionic environment using active transport processes to maintain a preferred cellular membrane potential [59]. Active transport processes often require chemical energy, and concomitant supplemental ATP generating processes is often activated to supply ATP needed for active ion extrusion [60]. ATP generation during osmostress has been detailed in *Synechocystis* sp. PCC6803, where the ATP generation is coupled to photosynthesis [19].

Some bacteria synthesize metabolites, such as trehalose or glutamate, which remain in the cellular cytoplasm that serve to decrease the intracellular concentration of inorganic ions and maintain protein folding and function [59]. These synthesized counter-osmolytes typically do not interfere with cellular processes, and can also act as chaperones and promote correct protein folding and function [39].

Bacteria also adjust the fatty acid composition of their cell membranes to increase membrane fluidity and decrease chemical permeability, making cells less susceptible to the physical effects of osmotic pressure [30]. Typical membrane composition changes in response to elevated extracellular osmolarities are an increase in anionic membrane lipids [41], and cardiolipin [29].

The response to osmotic stress, beyond the observed effect on growth rate and product formation [9], has not been studied in the genus *Caldicellulosiruptor*, and it remains unclear which adaptation systems, if any, are utilized to counter the effects of elevated osmolarity. An adaptive evolutionary effort to evolve more osmoresistant strains was conducted, resulting in strains capable of growing in elevated concentrations of glucose and acetate [35]. The effects of osmolarity on growth and fermentation productivity were empirically modeled in the closely related species *Caldicellulosiruptor saccharolyticus* [27]. It was found that the effect of elevated osmolarity was secondary to that of the inhibitory effect of dissolved hydrogen during continuous growth at moderate dilution rate. Hydrogen can super-saturate in this cultivation system [27], and the *Caldicellulosiruptor* genus relies heavily on hydrogen production for ferredoxin turnover [53]. The growth conditions employed in these studies contained relatively low levels of dissolved osmolytes (e.g., 5 and 10 g/L initial glucose loading), and did not explicitly assess for osmotolerance across a broad range of osmolarities. Nonetheless, it was found that decreases in growth and product evolution become prominent at 0.2 Osm/L, agreeing with an earlier finding that batch growth rates of *C. saccharolyticus* are reduced by 60% at 0.218 Osm/kg water [57]. Actively growing cells are able to continue growth in 520 mOsm/L after being supplemented with additional ammonium, and are able to initiate growth in medium with a starting osmolarity > 550 mOsm/L after an extended lag phase [3], indicating strong growth inhibition. Interestingly, no growth was initiated in spent medium containing, among other osmolytes, 165 mM of organic acids produced during fermentation of carbohydrates. This disparity suggests *C. bescii* is differentially resistant to different osmolytes. Ions capable of permeating, or being actively transported across, cell membranes are typically more cytotoxic than otherwise charge-neutral osmolytes, as they can disrupt and decrease membrane potential which is otherwise converted to useful chemical potential energy for the cell [60].

A low osmolarity defined medium was developed to alleviate the effects on growth which occur when *C. bescii* was grown in other growth media [15]. The osmolarity of this LOD (low osmolarity defined) medium was half of that of previous media, made possible by eliminating yeast extract, and decreasing the concentrations of macronutrients, among other changes. It was found that more osmolytes could be added before equivalent growth defects were observed in this medium, relative to media used previously with *Caldicellulosiruptor* [15].

Tolerance to osmolarity is a complex trait, as it involves the coordinated action of multiple cellular subsystems. Engineering more complex traits is difficult and cumbersome when modifying components of each system involved separately. Modifying regulatory systems can potentially affect larger gene/metabolic space with fewer number of mutations than modifying enzymes, as modifying one global regulator can affect expression of many different operons charged with many different functions in a coordinated fashion [55]. Success has been had previously in engineering regulatory mechanisms of organisms, model and non-model alike, toward desired traits [16, 23, 50]. In this study, we engineer regulatory machinery of *C. bescii* to effect osmotolerance and characterize resulting strains using growth and systems-level experiments.

## Results

### Growth phenotypes of single-gene deletion strains of two regulatory proteins

Single-gene deletion strains of regulatory genes were assessed for performance differences in CBP-related growth conditions (Additional file 1: Figure S1). Regulatory gene targets were selected from genes displaying differential expression in either or both of two previous differential gene expression studies [4, 51]. Also considered in the selection of gene targets were genes which were collocated with (and putatively regulated by) candidate regulatory gene targets, and the hypothesized regulatory/signaling actions of candidate gene targets. Single-gene deletion strains were generated for each of the 10 candidate regulatory genes (Table 1) and these strains, along with their genetic parents, were assayed in eight different CBP relevant growth conditions. Growth conditions included various soluble and insoluble carbon sources, including non-pretreated, washed switchgrass, xylan, crystalline cellulose, glucose, or xylose. Other conditions which are representative of stress conditions encountered during CBP were assayed including addition of methyl viologen to simulate redox stress, added ethanol to simulate the stresses of fermentation product accumulation, and added NaCl to simulate the stress of elevated osmolarity.

**Table 1:** Strains used in this study

Strain	Genotype	Reference
ORCB001	ΔB5X54_RS06355 Δ*pyrFA*	This study
ORCB002	ΔB5X54_RS01260 Δ*pyrFA*	This study
ORCB003	ΔB5X54_RS05670 Δ*pyrFA*	This study
ORCB004	ΔB5X54_RS02215 Δ*pyrFA* Δ*cbeI*	This study
ORCB005	ΔB5X54_RS11065 Δ*pyrFA*	This study
ORCB006	ΔB5X54_RS12050 Δ*pyrFA*	This study
ORCB007	ΔB5X54_RS07610 Δ*pyrFA* Δ*cbeI*	This study
ORCB008	ΔB5X54_RS04585 Δ*pyrFA*	This study
ORCB009	ΔB5X54_RS08965 Δ*pyrFA* Δ*cbeI*	This study
ORCB010	ΔB5X54_RS03805 Δ*pyrFA*	This study
JWCB005	Δ*pyrFA*	This study
JWCB018	Δ*pyrFA* Δ*cbeI*	This study
ORCB011	ΔB5X54_RS01260 Δ*pyrFA* pJGW07::Kan	This study
ORCB012	ΔB5X54_RS01260 Δ*pyrFA* pJGW07::B5X54_RS01260::Kan	This study
ORCB013	ΔB5X54_RS01260 Δ*pyrFA* pJGW07::B5X54_RS06355::Kan	This study

**Fig. 1:**
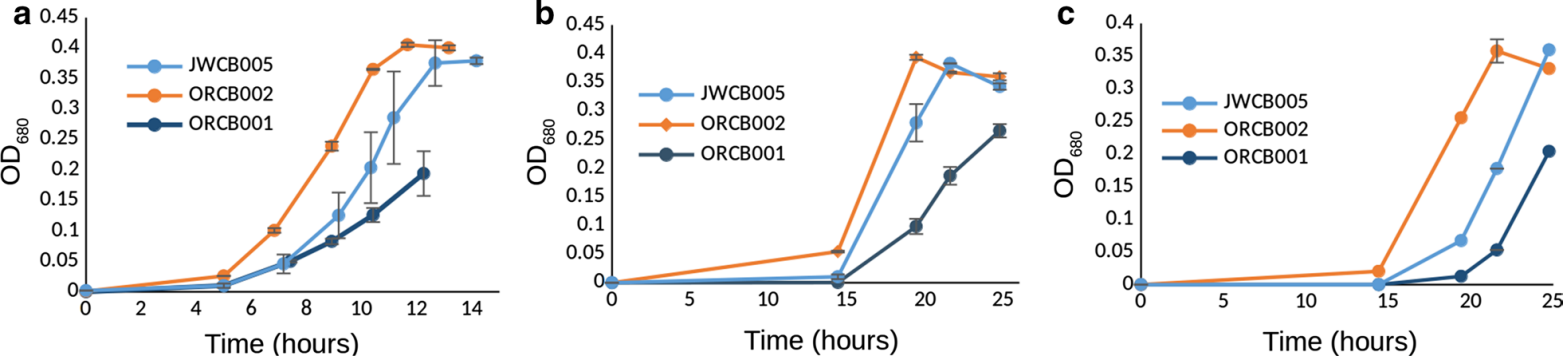
Growth phenotypes of strains ORCB001 and ORCB002 when grown in increasing amounts of added glycerol. Glycerol was added to make total initial (calculated) media osmolarities of **a** 400 mOsm/L, **b** 500 mOsm/L, and **c** 600 mOsm/L. Strain JWCB005 is the genetic parent strain to both single-gene deletion strains. Growth assays were done in 50 mL culture volumes in 135 mL serum bottles containing a headspace of 100% N_2_

**Fig. 2:**
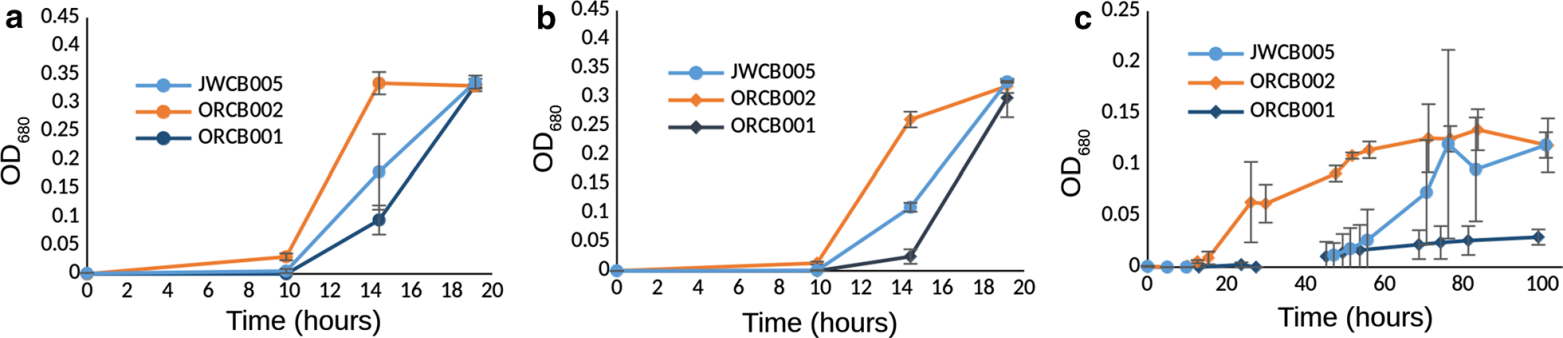
Growth phenotypes of strains ORCB001 and ORCB002 when grown in increasing amounts of added NaCl. NaCl was added to make total initial (calculated) media osmolarities of **a** 200 mOsm/L, **b** 300 mOsm/L, and **c** 400 mOsm/L. Strain JWCB005 is the genetic parent strain to both single-gene deletion strains. Growth assays were done in 50 mL culture volumes in 135 mL serum bottles containing a headspace of 100% N_2_

**Fig. 3:**
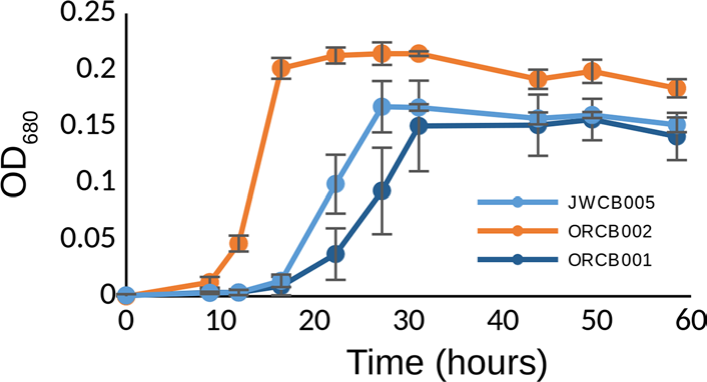
Growth phenotypes of strain ORCB001 and ORCB002, and their common genetic parent strain JWCB005, when grown in liquid culture containing 20 g/L added ethanol. Growth assays were done in 10 mL culture volumes in 26 mL Balch tubes containing a headspace of 10% CO_2_, 5% H_2_, and the balance N_2_

**Fig. 4:**
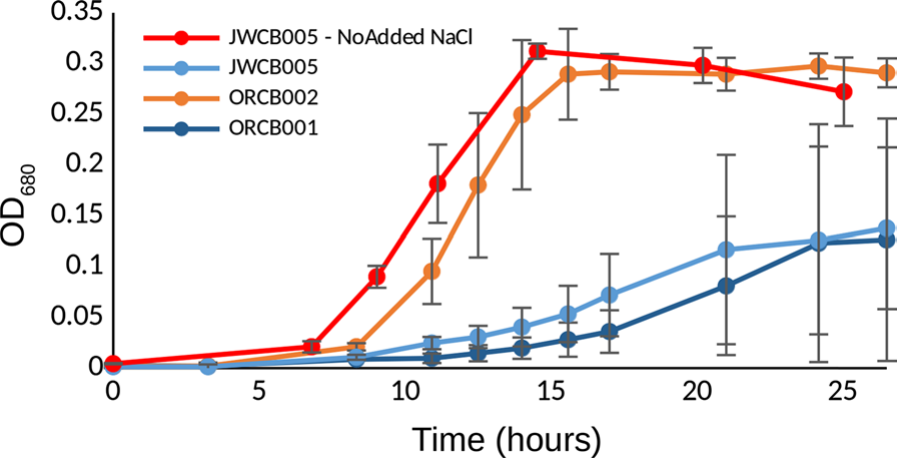
Growth phenotypes of strain ORCB001 and ORCB002, and their common genetic parent JWCB005, when grown in liquid culture containing added NaCl to make calculated initial osmolarity of 200 mOsm/L. Red line/markers indicate JWCB005 grown in medium containing no added NaCl. Growth assays were done in 10 mL culture volumes in 26 mL Balch tubes containing a headspace of 10% CO_2_, 5% H_2_, and the balance N_2_

Across the ten deletion strains and conditions screened for growth and fermentation phenotypes, we found two single-gene deletion strains which both displayed prominent growth phenotypes when assayed under various elevated osmolarity conditions (glycerol, NaCl, ethanol, Figs. 1, 2, 3, respectively). Glycerol was used as an osmolyte to contrast with NaCl because of their chemical differences and not being main substrates for fermentation. When NaCl was added to the growth medium to make the total starting osmolarity 200 mOsm/L (calculated), strain ORCB002 displayed an estimated growth rate of 0.4/h, while its genetic parent displayed a similarly estimated growth rate of 0.12/h in the same medium/condition (Fig. 4). At a starting osmolarity of 400 mOsm/L (achieved by adding NaCl), strain ORCB002 was in lag phase for 15 h prior to initiating log-phase growth, while its genetic parent (JWCB005), after inoculation of equivalent number of cells, was in lag phase for 47 h (Fig. 2). We attribute subtle growth differences between Figs. 2a and 4 to different assay formats (i.e., tube or bottle and possible differences in factors such as partial pressure of CO_2_), although note relative growth differences between the strains is similar between the two assays and in RNA-Seq studies (see Additional file 2: Figure S2a). Similar growth phenotypes were observed in elevated osmolarity media made with glycerol (Fig. 1) and ethanol (Fig. 3). In contrast to the improved growth phenotype observed in the Δ*cra* strain (ORCB002), strain ORCB001 displayed more severe growth defects than its genetic parent strain (JWCB005) across increasing concentrations of added NaCl, glycerol, or ethanol (Figs. 1, 2, and 3, respectively). Curiously, the relative growth defects that were observed when these strains were grown in LOD medium containing 20 g/L added ethanol (Fig. 3), were observed in 10 mL culture volumes in Balch tubes and 100% N_2_ headspace, and could not be replicated in 50 mL cultures contained in 135 mL serum bottles and a headspace of 5% H_2_, 10% CO_2_, and 85% N_2_.

**Fig. 5:**
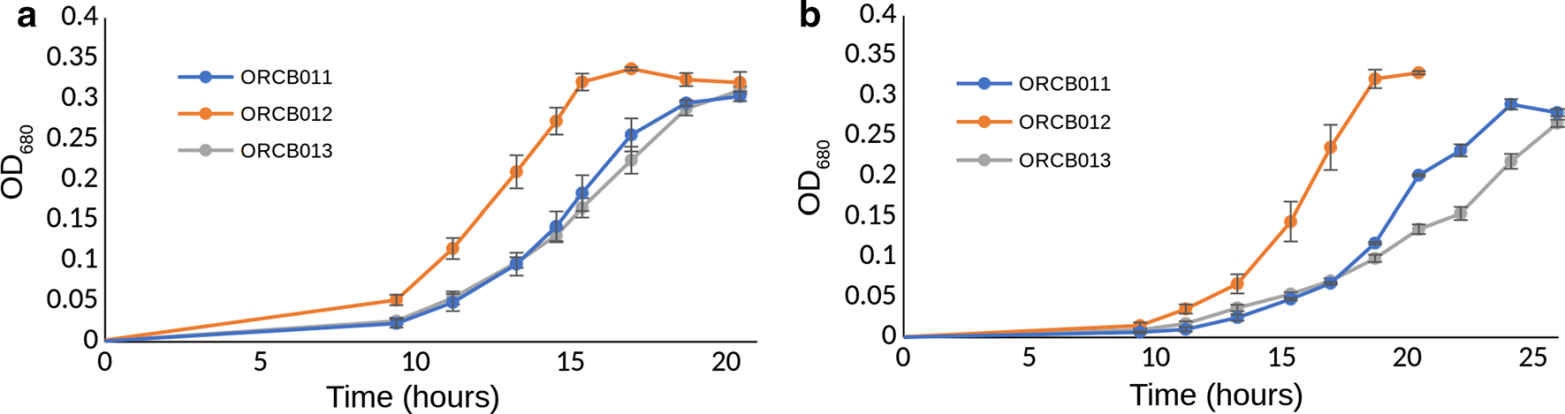
Growth phenotypes of strains ORCB011, ORCB012, and ORCB013 when grown in **a** added glycerol to total starting osmolarity of 500 mOsm/L, and **b** added NaCl to total starting osmolarity of 300 mOsm/L. The genetically complemented *fruR/cra*-deletion strain ORCB012 exhibited shorter lag phases and increased growth rates relative to an empty vector control (ORCB011), suggesting osmolyte phenotypes observed in ORCB002 are not due to the absence of the *fruR/cra* gene, but instead from other unintended genomic differences between ORCB002 and its genetic parent strain JWCB005

The complemented *fruR/cra*-deletion strain ORCB012 exhibited a shorter lag phases and higher growth rate than a *fruR/cra*-deficient strain with an empty expression vector (ORCB011) (Fig. 5). The lack of phenotype reversion to the slow-growth phenotype observed with the parental strain (JWCB005) suggests the absence of *fruR/cra* is not responsible, but the result of other unintended genetic mutations in ORCB002. Further, on the basis of growth rate and time spent in lag phase, a *fruR/cra*-deficient strain expressing the *fapR* gene (ORCB013) grew similarly to a *fruR/cra*-deficient strain with an empty vector (Fig. 5) in the presence of elevated osmolarity, suggesting under the conditions tested over-expressing the *fapR* gene was not a viable strategy for further enhancing osmotolerance in *C. bescii*.

### Proteomic assessment of ORCB012

A differential proteomic analysis of strain ORCB012, relative to its genetic parent strain ORCB002, confirmed the complemented strain successfully expressed the B5X54_RS01260 (FruR/Cra) protein product (Additional file 3: Table S2).

### Genome sequencing and variant detection of ORCB002

As the increased tolerance to osmolarity phenotype appeared to be stable and heritable in strain ORCB002, and genetic complementation did not revert the observed phenotype toward renewed sensitivity to increased osmolarity, we re-sequenced the genome of ORCB002 to identify other mutations which may be responsible for the apparent increased tolerance to osmolarity stress. It was discovered that, in addition to the deletion of the ΔB5X54_RS01260, a number of other genomic differences were observed between this strain and its genetic parent strain JWCB005.

Two regions of the genome incurred large deletions, comprising a total of 16 deletions that were either partial or complete (Table 2). Another gene incurred a mutation which resulted in a frameshift within its coding sequence (B5X54_RS01120). Two genes incurred mutations which introduced amino acid substitutions into their coding sequences (SAMN04515608_2488 and B5X54_RS08370), and another gene exhibited a SNP which resulted in a silent mutation (B5X54_RS07295). One large region of gene loss found in the ORCB002 strain (B5X54_RS07480–B5X54_RS07510) are genes affiliated with uracil auxotrophy in *C. bescii* and, as such, the selection and counter-selection strategies used to generate these mutants. These results confirm the genetic alteration suspected from the inability to obtain a PCR product from this region of expected length when the genome of this strain was used as template (Additional file 2: Figure S2). Adjacent genes which were also missing from this genomic location were two HAAT family amino acid/amide transporters, B5X54_RS07505 (full CDS deletion) and B5X54_RS07510 (partial CDS deletion).

**Table 2:** Genomic variants observed in strain ORCB002

Locus tag	Annotation	Putative homologs *in C. bescii* JWCB005 genome	Resulting mutation
B5X54_RS04240	CopG antitoxin of type II toxin-antitoxin system	No	Deletion/missing
B5X54_RS04245	Hypothetical protein	No	Deletion/missing
B5X54_RS04250	Hypothetical protein	WP_015907537.1	Deletion/missing
B5X54_RS04255	Hypothetical protein	WP_015907536.1	Deletion/missing
B5X54_RS04260	Hypothetical protein	WP_015907535.1	Deletion/missing
B5X54_RS04275	XRE family transcriptional regulator	No	Deletion/missing
B5X54_RS04280	DNA primase	No	Deletion/missing
B5X54_RS04285	protein of unknown function, DUF 3987	WP_015907299.1	Deletion/missing
B5X54_RS04290	Site-specific recombinase XerD	No	Deletion/missing
B5X54_RS07480	Dihydroorotate dehydrogenase electron transfer subunit	No	Partial deletion
B5X54_RS07485	Dihydroorotate dehydrogenase (NAD+) catalytic subunit, pyrD	No	Deletion/missing
B5X54_RS07490	Probable phosphoglycerate mutase	No	Deletion/missing
B5X54_RS07495	Orotate phosphoribosyltransferase, pyrE	No	Deletion/missing
B5X54_RS07500	Double zinc ribbon	No	Deletion/missing
B5X54_RS07505	Amino acid/amide ABC transporter ATP-binding protein 2, HAAT family	No	Deletion/missing
B5X54_RS07510	Amino acid/amide ABC transporter ATP-binding protein 1, HAAT family	No	Partial deletion
B5X54_RS01120	ABC-2 type transport system ATP-binding protein	No	Frameshift
B5X54_RS07295	Protein of unknown function	No	Silent mutation
B5X54_RS08370	Molecular chaperone DnaK	No	Substitution
SAMN04515608_2488, no analogous locus tag	Carbohydrate ABC transporter substrate-binding protein, CUT1 family	WP_105908637.1, WP_099716409.1	2 Adjacent substitutions

Homologs were determined as > 90% amino acid identity while utilizing > 90% locus tag amino acid sequence for alignment

Another large region of deletion comprised two non-contiguous regions (B5X54_RS04240-B5X54_RS04260 and B5X54_RS04275-B5X54_RS04290) between which two genes reside; a hypothetical gene and an RNA polymerase sporulation sigma factor; sigma 70 (B5X54_RS04265 and B5X54_RS04270). Interestingly, these two genes as well as 1343 bp downstream of these genes are also present as contiguous sequence at one other location in the genome and on the native plasmid pATHE01 in the *C. bescii* DSM6725 genome. The missing genes upstream of these two genes comprise four unannotated genes and a CopG antitoxin of type II toxin–antitoxin system. The genes which are not present downstream of these two genes contain an XRE family transcriptional regulator, and a XerD site-specific recombinase and another unannotated gene. There exist apparent homologs (> 90% coverage and > 90% protein identity similarity from a BLASTp homology search) of three of the four unannotated genes (B5X54_RS04250, B5X54_RS04255, and B5X54_RS04255) elsewhere on the genome of strain ORCB002.

It was found that a gene encoding the *dnaK* molecular chaperone (B5X54_RS08370) contained one non-synonymous SNP (T516A), while a gene encoding a carbohydrate ABC transporter substrate-binding protein, CUT1 family contained two non-synonymous SNPs (A28V and T27A) that are at adjacent positions in the amino acid sequence.

### Transcription factor binding site prediction

As part of the characterizing the transcriptional regulatory systems we examined putative binding sites using the Virtual Footprint tool associated with the PRODORIC database [33], The *C. saccharolyticus* genome was used for individual FruR binding sites with the consensus binding site predicted in *Thermotoga* [34]. These searches did not yield any putative binding sites. Manual inspection of the *Thermotoga* FruR binding sites predicted in RegPrecise [34] suggests the sites predicted in this database for this organism are about twice the length of typical FruR/Cra transcription factor binding site predicted in other organisms (~ 18–20 bp). This led us to hypothesize that the *Thermotoga* consensus binding sites identified in the RegPrecise database may actually be two individual FruR/Cra binding sites oriented adjacent to each other. Splitting these two previously predicted binding sites in half yielded four ‘half sites’ with the regular expression consensus sequence (A|G)TCATAA(A|T)NNNNNAT(A|C)ANN; characterized by an AT-rich 3′ sequence. This consensus sequence is similar in length and the conserved position sequence predicted for the FruR/Cra transcription factor in other organisms curated in the RegPrecise database [34]—particularly those genera in which consensus binding sites have been compiled using far more individual binding sites, such as *Bacillus* (17 predicted binding sites) and *Streptococcus* (34 predicted binding sites). The only putative site identified immediately outside of coding regions of the *C. saccharolyticus* genome, when searching for this manually generated consensus sequence, was a site located 46 bp immediately upstream of the *fruR*/*cra* gene itself. This presumably indicates local negative auto-regulation of the *fruR*/*cra* gene and other collocated genes contained in the same putative transcriptional unit. Similarly, we were able to identify FapR binding sites in the *C. bescii* JWCB005 genome that are homologous to previously predicted FapR binding sites in *C. saccharolyticus* [4]; upstream of B5X54_RS06355 (the first gene in a putative fatty acid biosynthesis transcription unit) and B5X54_RS07175 (the first gene in a putative malonyl-CoA biosynthesis transcription unit).

### Direct and indirect regulatory actions of FapR and Cra in *C. bescii*

In order to provide a more complete assessment of the regulons of these two transcription factors, we conducted RNAseq analysis of these two deletion strains, and their common genetic parent strain, under three different moderately elevated osmolarity conditions (added glycerol to total 500 mOsm/L, added glucose to total 300 mOsm/L, added NaCl to total 300 mOsm/L, Additional file 4: Figure S3).

Genes contained in putative operons encoding genes responsible for fatty acid biosynthesis (B5X54_RS06360–B5X54_RS06395) and malonyl-CoA biosynthesis (B5X54_RS07175–B5X54_RS07190) displayed increased expression in strain ORCB001 (Table 3).

**Table 3:** Genes in strain ORCB001 which exhibit increased expression when cells were cultured in elevated osmolarity conditions

Locus tags	Annotation	log_2_ (ORCB001/JWCB005)		
		Glycerol	Glucose	NaCl
**B5X54_RS06360**	Phosphate acyltransferase	3.9	2.9	2.8
**B5X54_RS06365**	Ketoacyl-ACP synthase III	3.8	2.3	2.2
**B5X54_RS06370**	Malonyl CoA-acyl carrier protein transacylase	3.8	2.5	2.2
**B5X54_RS06375**	Beta-ketoacyl-ACP reductase	3.7	2.2	2.1
**B5X54_RS06380**	Acyl carrier protein	2.1	1.5	1.3
**B5X54_RS06385**	Beta-ketoacyl-[acyl-carrier-protein] synthase II	2.8	1.8	1.7
**B5X54_RS06390**	Ribonuclease III	2.2	1.3	1.3
**B5X54_RS06395**	Radical SAM protein	2.3	1.3	1.5
*B5X54_RS07175*	Methylmalonyl-CoA carboxyl transferase	3.6	1.6	1.9
*B5X54_RS07180*	Hypothetical protein	3.6	1.8	2.2
*B5X54_RS07185*	Acetyl-CoA carboxylase biotin carboxyl carrier protein subunit	3.6	1.8	2.1
*B5X54_RS07190*	Oxaloacetate decarboxylase	3.7	1.7	2.0

Colors denote adjacent genes which may be co-expressed as part of the same transcriptional unit. Genes denoted in bold are responsible for fatty acid biosynthesis. Genes denoted in italics are responsible for the metabolism of malonyl-CoA, a fatty acid biosynthesis precursor and the regulatory effector metabolite which binds the FapR transcription factor. All values are statistically significant (Wald test, Benjamini–Hochberg adjusted *p* value < 0.05, *n* = 3)

In strain ORCB002, increased expression was observed in genes involved in fructose PTS as well as a phosphofructokinase (B5X54_RS01265–B5X54_RS01290) (Table 4), genes which are also collocated with the B5X54_RS01260 gene. Interestingly, we find these genes to not be significantly differentially expressed under elevated levels of glucose, suggesting these genes may be differentially regulated in response to glucose as compared to other osmolytes assayed. Additionally, two adjacent genes (a transposase, sigma factor E) were differentially expressed in strain ORCB002 when grown in elevated levels of glucose and glycerol, but not NaCl, and a gene encoding sigma factor G (B5X54_RS04530) differentially expressed under all three elevated osmolarity conditions tested (Table 4).

**Table 4:** Genes in strain ORCB002 which exhibit increased expression when cells were cultured in elevated osmolarity conditions

Locus tag	Annotation	log_2_(ORCB002/JWCB005)		
		Glycerol	Glucose	NaCl
**B5X54_RS01265**	1-Phosphofructokinase	4.2	0.8	4.5
**B5X54_RS01270**	PTS fructose transporter subunit IIA	4.5	0.7	4.5
**B5X54_RS01275**	PTS fructose transporter subunit IIBC	4.8	0.6	4.3
**B5X54_RS01280**	HPr family phosphocarrier protein	4.1	0.8	3.7
**B5X54_RS01285**	Phosphoenolpyruvate-protein phosphotransferase	4.5	0.6	4.0
**B5X54_RS01290**	Acetylesterase	2.4	0.4	2.7
*B5X54_RS04520*	Transposase	1.4	1.8	0.6
*B5X54_RS04525*	Sporulation sigma factor SigE	1.5	1.4	0.9
*B5X54_RS04530*	Sporulation sigma factor SigG	1.3	1.2	1.0

Bold and italics denote adjacent genes which may be co-expressed as part of the same transcriptional unit. Bold text denotes statistical significance (Wald test, Benjamini–Hochberg adjusted *p* value < 0.05, *n* = 3). Values that are not statistically significant are denoted in underline

We also find genes displaying decreased expression levels in strain ORCB002 (Table 5). Three adjacent genes (B5X54_RS01990–B5X54_RS02000) showed similar differential expression patterns. B5X54_RS01990 and B5X54_RS01995 are annotated as hypothetical proteins, and B5X54_RS02000 is annotated as a peptidase S8 protein. Similarly, a vaguely annotated oxidoreductase gene, B5X54_RS07480, showed decreased expression in all three elevated osmolarity conditions.

**Table 5:** Genes in strain ORCB002 which exhibit decreased expression when cells were cultured in elevated osmolarity conditions

Locus tag	Annotation	log_2_(ORCB002/JWCB005)		
		Glycerol	Glucose	NaCl
**B5X54_RS01990**	Hypothetical	− 3.3	− 2.2	− 2.9
**B5X54_RS01995**	Hypothetical	− 3.5	− 2.9	− 3.2
**B5X54_RS02000**	Peptidase S8	− 3.8	− 3.7	− 3.5
*B5X54_RS07480*	Oxidoreductase	− 1.3	− 2.2	− 1.6

Bold and italics denote adjacent genes which may be co-expressed as part of the same transcriptional unit. All values are statistically significant (Wald test, Benjamini–Hochberg adjusted *p* value < 0.05, *n* = 3)

Three genes exhibited decreased expression in both strains; B5X54_RS00980, B5X54_RS05305, and B5X54_RS05310 (Table 6). The latter two are annotated as hypothetical proteins, adjacent to each other and may likely be part of the same transcriptional unit. B5X54_RS00980 is annotated as an endo-1,4 beta xylanase. As these genes showed similar expression patterns in two strains (ORCB001 and ORCB002) which displayed largely different relative growth phenotypes, it is further unlikely they are contributing to the observed phenotypes and were not considered further. Additional genes showed significantly decreased expression in strain ORCB002. These genes were distinct from other genes displaying differential expression in that their average normalized read count values across collected samples were near zero (Table 7). It is confirmed through our genomic resequencing analysis (Table 2) that this observed differential expression is artifactual carryover from genomic alterations which have occurred in the strain. One such example is a locus containing many genes involved in uracil biosynthesis and sits adjacent to the *pyrF* gene (B5X54_RS07475) which is truncated and non-functional in this strain [8]—the truncation making uracil-auxotrophy based genetic modifications possible. PCR amplification of a region spanning the genomic region between genes B5X54_RS07485 and B5X54_RS07515 resulted in a single product of expected size when using genomic DNA from strain JWCB018 (an auxotrophic derivative of strain JWCB005) as template, though no PCR product could be amplified when using genomic DNA from ORCB002 as PCR template (Additional file 2: Figure S2). Furthermore, repeated efforts to transform strain ORCB002 using *pyrF* as a positive heterotrophic selection marker did not yield any transformants, suggesting the additional genes necessary for uracil biosynthesis that are missing in this strain are contributing to uracil auxotrophy. Differential expression from these genes, and other genes showing normalized read count values < 5 were omitted from our analysis (Table 7).

**Table 6:** Genes that were found to have similar differential expression in strains ORCB001 and ORCB002, each compared to their common genetic parent strain JWCB005

Locus tag	Annotation	Average log_2_(ORCB002/JWCB005) across three osmolarity conditions	Average log_2_(ORCB001/JWCB005) across three osmolarity conditions
**B5X54_RS00980**	Endo-1,4-beta xylanase	− 4.1	− 4.2
*B5X54_RS05305*	Hypothetical	− 4.9	− 2.5
*B5X54_RS05310*	Hypothetical	− 4.3	− 2.4

Values are average differential log-fold changes observed for the indicated gene across three indicated high-osmolarity conditions (glucose, glycerol, sodium chloride). As these genes were similarly differentially expressed in strains showing different growth phenotypes, it is unlikely these genes are contributing to observed phenotype differences, and were not considered in this study. All values are statistically significant (Wald test, Benjamini–Hochberg adjusted *p* value < 0.05, *n* = 3)

**Table 7:** Genes found to have relatively low normalized expression values in strain ORCB002

Locus tag	Annotation	log_2_(ORCB002/JWCB005)			Average normalized read counts		
		Glycerol	Glucose	NaCl	ORCB002	JWCB005	ORCB001
**B5X54_RS04240**	DNA-binding protein	− 6.6	− 6.4	− 6.8	0.58	366	346
*B5X54_RS04275*	Transcriptional regulator	− 6.5	− 7.5	− 7.8	0.57	659	773
*B5X54_RS04280*	DNA primase	− 6.9	− 7.5	− 8.3	0.57	824	968
*B5X54_RS04285*	Hypothetical protein	− 7.1	− 7.5	− 7.7	0.57	856	828
*B5X54_RS04290*	Site-specific integrase	− 6.6	− 5.6	− 6.2	0.12	300	277
*B5X54_RS04295*	tRNA-Pro	− 5.6	− 5.2	− 5.7	1.6	212	220
B5X54_RS07485	Dihydroorotate dehydrogenase	− 6.6	− 6.9	− 6.8	0.59	358	383
B5X54_RS07490	Histidine phosphatase family protein	− 6.5	− 6.6	− 7.0	0.62	350	401
B5X54_RS07495	Orotate phosphoribosyltransferase	− 6.8	− 6.9	− 6.9	1	467	523
B5X54_RS07500	Hypothetical protein	− 6.6	− 6.4	− 6.6	0.39	290	304

Many of these very low expression values are artefactual, and instead the result of genomic alterations in this strain (as confirmed by genome resequencing of the ORCB002 strain displayed in Table 2). All differential expression log_2_ values are statistically significant (Wald test, Benjamini–Hochberg adjusted *p* value < 0.05, *n* = 3)

## Discussion

Substantial growth phenotypes were exhibited by two single-gene deletion strains of *C. bescii*. A single-gene deletion of the *fapR* gene exhibited a considerable growth defect when grown in elevated levels of osmolarity (Figs. 1, 2, 3, 4) and strain ORCB002 grew substantially better than its genetic parent strain (on the basis of growth rate and time in lag phase) in elevated levels of added osmolarity (Figs. 1, 2, 3, 4). Neither strain displayed growth phenotypes when grown in unaltered, replete LOD medium (data not shown).

### Complementation analysis and proposed mechanism of FapR and Cra-enabled osmotolerance in *C. bescii*

We confirm that the FapR transcription factor does regulate fatty acid biosynthesis and malonyl-CoA metabolism genes in *C. bescii*, as predicted previously for the closely related species *C. saccharolyticus* [4]. Over-expressing the *fapR* gene did not, however, yield a strain exhibiting superior osmotolerance (Fig. 5). Complementation analysis and differential proteomic analysis confirmed successful homologous expression of the Cra/FruR protein from plasmid pJGW07::B5X54_RS01260::Kan in the genetically complemented strain, which suggests it may not be the deletion of the *cra* gene that is the cause of observed growth phenotypes in strain ORCB002. Unintended genetic differences leading to phenotypes have been observed previously in *C. bescii*, and the species is thought to have a highly active transposase capable of inactivating genes and causing genome alterations [56].

Resequencing analysis of strain ORCB002 revealed several genomic alterations which could be responsible for its increased ability to tolerate elevated osmolarity relative to its genetic parent. Our SNP/variation mapping did not find any additional copies, or alterations otherwise in the existing loci of the ISCbe4 transposable elements [11, 56] in strain ORCB002, relative to that of its parent strain JWCB005. Of the genes that were found to be deleted or mutated (Table 2), only the *dnaK* chaperone has been previously implicated in affecting tolerance to increased levels of osmolarity [32, 49]. However, the residue at which the SNP is present in this gene strain has not been previously studied to our knowledge, and it remains unclear from these investigations what effect the substitution introduced in this gene (T516A) has on the in vivo activity of this gene or the expression levels of this gene in this strain.

Genomic alterations suspected in the pyrimidine biosynthesis pathway of ORCB002 were confirmed by whole-genome resequencing. It has been observed previously [56] that disruptions in other genes at this locus can effectively serve as selection markers by preventing strains from synthesizing uracil de novo. One of the genes missing at this locus in strain ORCB002 is the same gene (*pyrE*) a genes whose deletion endowed the strain with 5-FOA resistance and made the strain amenable to uracil/5-FOA selection/counterselection genetics [25].

### Improving osmoresistance in *C. bescii* biocatalysts through regulatory metabolic engineering

Osmosensitivity is one of the defining needs in a candidate industrial CBP organism. To make 50 g/L ethanol, a nominal target for consolidated bioprocessing, the final fermentation osmolarity will exceed that of seawater (which is ~ 1000 mOsm/L) and a CBP biocatalyst will need to actively ferment at these osmolarities. The cellular response to osmotic shock involves a large number of cellular sub-processes [59], many of which are poorly understood in *C. bescii*. We affected maximal gains in osmoresistance in *C. bescii* by making very few genetic modifications, though further complementation analysis of one strain suggests that unintended genetic alterations may be responsible for the osmoresistance phenotype observed in strain ORCB002. Engineering a more osmoresistant strain using only rational metabolic engineering would, at present, be difficult without further characterizing osmolarity response systems in *C. bescii*, and the effects of the other mutations identified in strain ORCB002.

### Osmolarity stress response and the cellular function of the FapR and FruR/Cra transcription factors in *C. bescii*

FapR is a conserved transcription factor present in Gram-positive bacteria. Its regulon consists of the genes responsible for fatty acid biosynthesis as well as malonyl-CoA metabolism [46]. Malonyl-CoA also serves as the binding cofactor molecule for FapR. It has been shown previously in other organisms that altering this transcription factor can affect cell growth [26], the composition of cellular membrane lipids [47, 36], and affect tolerance to osmotic stress [18]. The *fapR* gene, and associated regulon genes, was shown to be upregulated under hydrogen sparging in the closely related species *C. saccharolyticus* [4], though it remains unknown if this condition presents an osmotic challenge to *C. saccharolyticus*. In this study we demonstrate that the *fapR* gene likely regulates genes involved in malonyl-CoA and fatty acid biosynthesis, as it does in a conserved fashion in many other bacteria. The *fruR* gene was first characterized and shown to be a local regulator of fructose metabolism and the fructose-specific phosphotransferase system (PTS), and was subsequently shown to have a regulon extending far beyond just genes it is collocated with [37, 38, 43]. Upon discovery of this expanded regulon, the gene was aptly renamed Cra (for cyclic AMP independent repressor/activator). Cra was shown to both activate genes as well as repress genes [38], as well as work coordinately with other carbon regulatory genes to globally regulate carbon metabolism [42]. This transcription factor regulates expression in response to binding fructose 1-phosphate [6, 38], a metabolic intermediate in fructose metabolism. It is known to be a global regulator of carbon metabolism, regulating genes involved in fermentation, glycolysis/gluconeogenesis, carbohydrate transport, and the TCA cycle [5, 13, 38, 42]. Not surprisingly, the transcription levels of *fruR/cra* in *C. saccharolyticus* were increased when cells were grown on fructose [52], suggesting that this gene is in fact locally regulating genes contained within the same operon as *fruR/cra* which are responsible for fructose metabolism. A FruR/Cra homolog in *Thermotoga* shows 70–80% operon-level sequence homology with the *C. bescii* gene B5X54_RS01260 [40] and is predicted to be a local repressor of fructose metabolism genes, though neither this gene or its regulon have been biochemically or genetically or biochemically evaluated in *Thermotoga*.

We present putative evidence of local regulation by Cra in *C. bescii*, as *fru* operon genes appear to be coordinately expressed differently in high concentrations of glucose (Table 4). Though conserved homologs appear in many organisms [2, 7, 17, 40], it is unclear whether this gene plays a global regulatory role in *C. bescii*. Two findings support a global regulatory role of Cra in *C. bescii*; differential expression of two sigma factors were observed in RNAseq analysis of strain ORCB002 (Table 4), and differential protein abundance of other known transcription factors or genes belonging to regulatory gene families (highlighted in yellow, Additional file 3: Table S2) in proteomic abundance in strain ORCB012. The genes whose transcription is affected by these sigma factors in *C. bescii* remains unknown, though homologs of these two sigma factors are known to be active in stress-response [14, 22]. The RefSeq gene annotations of these two sigma factors annotate these genes as being involved in sporulation, though *C. bescii* is not known to form spores [20]. As both increased and decreased differential expression are observed in strain ORCB002 (Tables 4 and 5), this gene may have a pleiotropic regulatory role in *C. bescii*. From the findings in this work, it is not possible to determine if such pleiotropy would be the effect of direct or indirect regulatory action by this transcription factor.

## Conclusions

Through screening of ten single-gene deletion mutants of selected regulatory genes, we isolated two strains exhibiting phenotypically differential resistance to elevated, inhibitory levels of osmolarity in *C. bescii*. One strain carries a single-gene deletion of the FapR repressor, and the other a single-gene deletion of a gene that is most likely either the FruR local repressor of fructose metabolism or the global carbon metabolism regulator Cra. We engineered a *cra*-deficient strain of *C. bescii* that over-expressed the *fapR* gene with the hope of generating a highly osmotolerant strain of *C. bescii*. Unfortunately, the resulting strain did not exhibit further enhanced growth properties in elevated osmolarity conditions. Further, it is unlikely that the *cra* gene impacts osmotolerance in *C. bescii*, as complementation analysis of a *cra*-deficient mutant did not revert observed phenotypes of the *cra*-deficient strain. Other unintended genomic differences in this strain were identified through strain re-sequencing, and may be responsible for its growth performance in elevated osmolarity conditions.

Differential expression and prediction of transcription factor binding sites along the genome show the FapR gene regulates fatty acid biosynthesis genes as well as genes responsible malonyl-CoA metabolism, each organized into a functionally related putative transcriptional unit. The same analysis suggests FruR/Cra transcription factor negatively regulates a functionally related transcription unit containing genes responsible for fructose metabolism and fructose-specific PTS. In addition, differential expression and differential protein abundance evidence also suggests FruR/Cra has a regulatory effect on other genes, suggesting this transcription factor may play a global regulatory role in *C. bescii*, as it does in other bacteria.

## Methods

### Strain construction

Strains used in this study (Table 1) were constructed as described previously [10]. In an effort to generate a strain more osmotolerant than ORCB002, we overexpressed the *fapR* gene in this strain, resulting in strain ORCB013. Simultaneously, we conducted complementation analysis of strain ORCB002, using strain ORCB012, to assess whether the absence of the *fruR/cra* gene was, in fact, a cause of the phenotypes observed in the *fruR/cra*-deficient strain in elevated osmolarity conditions.

### Screening single-gene deletion mutants and expression mutants

Markerless deletions of 10 regulatory genes were generated in one of two strains of *C. bescii* (Table 1) using previously described methods [10, 11]. Single-gene deletion strains were grown in eight different conditions and screened for growth or fermentation productivity phenotypes. Media were prepared as described previously [15]. Initial screening cultures were all grown in Balch tubes containing 10 mL of medium. Cultures containing insoluble carbon substrates (xylan, crystalline cellulose, or switchgrass) were grown in 50 mL of medium in 135 mL serum bottles. All media contained 5 g/L of the respective carbon substrate. Cultures containing added methyl viologen, ethanol, or NaCl contained 5 g/L glucose as the primary source of carbon. Methyl viologen was utilized at a concentration of 150 mg/L, ethanol was added to a final concentration 20 g/L, and NaCl was utilized to make medium with an initial calculated total osmolarity of 200 mOsm/L. All media osmolarities were calculated based on accounting for media osmolarity of LOD medium reported previously [15], and adding osmolytes as dry chemicals sufficient to make each medium the desired osmolarity. All screening cultures contained a headspace of 100% N_2_. Growth and fermentation products were assayed as previously described [45]. Growth was monitored in cultures containing xylan or crystalline cellulose as the primary source of carbon by assaying total culture protein. To assay total culture protein (as a proxy for cell biomass in cultures utilizing crystalline cellulose or xylan as sole carbon sources), 300 µL of culture was added to 100 µL of 500 mM NaOH and incubated at 100 °C for 10 min. Incubated samples were then centrifuged (13,000×*g*, 10 min). Aliquots of the supernatant were analyzed using the Coomassie (Bradford) Protein Assay Kit (ThermoFisher Scientific catalog #23200) according to manufacturer’s instructions.

Subsequent osmolarity stressor assays (Figs. 1 and 2), as well as batch growth for RNAseq analysis (Figure S3), were done in 50 mL culture volumes, in elevated osmolarity conditions indicated, in 135 mL serum bottles containing a headspace of 5% H_2_, 10% CO_2_, and 85% N_2_. Stability of expression plasmids in expression strains was assessed by PCR amplification of the plasmid-borne expression cassette using 10X concentrated liquid culture as PCR template. Expression strains (and the exogenous plasmids they bore) were determined to be genetically stable if this PCR product was present after three transfers in selective liquid medium. Further, the presence of the plasmid expression cassettes were assessed after 30 h of fermentation from selected replicates of growth conditions during fermentation under elevated osmolarities (Fig. 5).

### Genome resequencing of ORCB002

The whole genome of strain ORCB002 was resequenced and mapped to the genetic parent strain (JWCB005) to ascertain genetic variations which may be responsible for its apparent increased tolerance to osmolarity. Whole cells were grown in LOD medium containing uracil and genomic DNA was extracted using a Qiagen Dneasy PowerLyzer PowerSoil Kit (Qiagen, Hilden, Germany) following the manufacturer’s protocol. Cells were lysed for 45 s at 6200 rpm on a Precellys 24 high-throughput tissue homogenizer (Bertin Technologies, Montigny-le-Bretonneux, France). DNA quality was assessed by NanoDrop (ThermoFisher Scientific, Waltham, MA) and quantity was determined by Qubit broad range double stranded DNA assay (ThermoFisher Scientific Invitrogen Waltham, MA). Nextera XT sequencing libraries (Illumina, San Diego, CA) were prepared according to the manufacturer’s recommendations stopping after library validation. The final library was validated on an Agilent Bioanalyzer (Agilent Technologies, Santa Clara, CA) using a DNA7500 chip and concentration was determined on a Qubit (ThermoFisher Scientific Invitrogen, Waltham, MA) with the broad range double stranded DNA assay. The library was prepared for sequencing following the manufacturer’s recommended protocol (15039740v09, Standard Normalization). One paired end sequencing run (2 × 251) was competed on an Illumina MiSeq instrument (Illumina, San Diego, CA) using a Nano v2 chemistry kit.

Sequencing reads were deposited into the NCBI Sequence Read Archive and are available through accession number PRJNA543682. Sequencing reads were mapped to the JWCB005 genome; NCBI GenBank accession/project number: FUZN00000000.1 (FUZN01.1), comprising 2 contigs; FUZN00000001.1 and FUZN00000002.1 using the ‘Map reads then find variations/SNPs’ workflow in the Geneious software (v. 11.1.5). In the ‘Map to Reference’ options of this workflow, the sensitivity was set to ‘High Sensitivity/Medium’ and all other settings were set to default. In the ‘Find Variations/SNPs’ options of this workflow, the ‘maximum variant *P*-value’ was set to 10^−4^, while all other settings were left as default. Resulting SNPs called were then further manually filtered based on the following thresholds; the variant frequency had to be present in > 90% of mapped reads, variations identified within tandem repeats longer than 5 nucleotides were omitted, only SNPs whose mapping coverage was greater than 27× were considered, and only reads whose average quality scores were > 30 were used to identify genomic variations.

### Proteomic determination of FruR/Cra expression in ORCB012

Strain ORCB012 was grown in LOD medium containing added uracil, and 50 mL of mid-log phase cell culture was collected, centrifuged, decanted and snap frozen in liquid nitrogen and stored at − 80 °C for further processing. Protein was isolated from cell biomass and prepared for bottom-up proteomics as described previously [48]. Peptide samples were analyzed by automated 1D LC–MS/MS analysis using a Vanquish UHPLC plumbed directly in-line with a Q Exactive Plus mass spectrometer (Thermo Scientific) outfitted with a trapping column coupled to an in-house pulled nanospray emitter. Both the trapping column (100 µm ID) and nanospray emitter (75 µm ID) were packed with 5 µm Kinetex C18 reversed phase resin (Phenomenex) to 10 cm and 30 cm, respectively. For each five-microgram sample inject, peptides were loaded, desalted, separated and analyzed across a 180 min organic gradient (240 min total considering load, desalting, and column re-equilibration steps). Eluting peptides were measured and sequenced by data-dependent acquisition on the Q Exactive mass spectrometer and resulting MS/MS spectra were searched against the *C. bescii* proteome concatenated with reversed entries (to assess false-discovery rates) and common protein contaminants using MyriMatch and IDPicker, as previously detailed [12]. Peptide abundances were extracted from chromatographic area-under-the-curve and summed to estimate protein-level abundance. Protein abundance distributions were then normalized across samples and compared across strains by pairwise *t* test.

### RNAseq analysis of single-gene deletion mutants under elevated osmolarity conditions

RNAseq analysis was conducted on strains grown to mid-log phase in three different elevated osmolarity conditions; added NaCl to a total initial osmolarity of 300 mOsm/L, added glycerol to make a total initial osmolarity of 500 mOsm/L, and added glucose to a total initial osmolarity of 300 mOsm/L. All cultures were grown to mid-log phase (Figure S3) at which point separate sacrificial 50 mL cultures were collected, centrifuged, and cell biomass pellets were snap frozen in liquid nitrogen. Growth was monitored in parallel-grown triplicate cultures until growth ceased (Additional file 4: Figure S3). RNA was extracted, and RNAseq libraries were synthesized and sequenced as described previously [44].

Fastq files were downloaded from servers at the contracted sequencing facility (HudsonAlpha) and the integrity was verified by computing their checksum and comparing these computed values to those provided by HudsonAlpha. Read quality was checked using FastQC (v. 0.11.5) (available online at https://www.bioinformatics.babraham.ac.uk/projects/fastqc/). RNAseq reads were trimmed the using Trimmomatic (v. 0.33) MAXINFO method with target length and strictness parameters set to 40 and 0.8, respectively. Reads were mapped to the genome of *Caldicellulosiruptor bescii* JWCB005 (GCF_900166995.1, last modified 2017/03/09) using Bowtie2 (v. 2.2.9) with the same parameters as the very sensitive preset option except the number of mismatches was set to 1. The number of successfully mapped reads for each gene in each sample were counted with HTSeq (v. 0.6.1p1). Differential expression was computed using the DEseq 2 R package using default parameters. Differentially expressed genes were determined to be those exhibiting a statistically significant log_2_ fold change of > 1 or < − 1, normalized average read counts above 50, and individual strain normalized read counts > 5 (Tables 3, 4, 5, 6). Raw, unprocessed RNAseq reads as well as differential expression data for all genes were deposited into the Gene Expression Omnibus database as accession number GSE107393.

### Transcription factor binding site prediction

Transcription factor binding sites were predicted for the FruR/Cra transcription factor using the *C. bescii* JWCB005 genome (NCBI Accession # PRJEB19583). A regular expression consensus binding site was adapted from *Thermotoga* FruR predicted binding sites in RegPrecise [34]; the sequence (A|G)TCATAA(A|T)NNNNNAT(A|C)ANN. This sequence was used to seed a transcription factor binding site sequence search using the Virtual Footprint Regulon Analysis tool [33]. The above-mentioned sequence was entered as ‘Regular Expression’ subpattern type and the maximum distance to gene (maximum promoter length) was set to 350 bp. The match properties ‘Ignore Match Orientation’ and ‘Remove Redundant (Palindromic) Matches’ were selected.

### Assessment of genome differences between JWCB005/JWCB018 and ORCB002

Primers were designed to amplify a genomic region spanning B5X54_RS07480–B5X54_RS07510; a primer was designed to anneal to the B5X54_RS07480 coding region (pyr fwd screen, Additional file 5: Table S1), and 3′ to the B5X54_RS07510 coding DNA sequence (pyr rev screen, Additional file 5: Table S1). Genomic DNA was extracted from the respective strains using a Zymo Research Quick-DNA Miniprep Kit (Catalog # D3006, Zymo Research Corp., Irvine, CA) according to manufacturer’s instructions, and used as template in PCR amplification carried out using Phusion 2X Master Mix according to manufacturer’s instructions (Catalog # M0531S, New England Biolabs, Ipswich, MA) (Additional file 6: Table S3).

## Supplementary information

**Additional file 1: Figure S1.** Growth or fermentation product analysis of 10 strains considered in this study in various growth conditions relevant to consolidated bioprocessing performance. a) Acetate supernatant concentrations in fermenting cultures of C. bescii grown in medium containing unpretreated switchgrass as the sole source of carbon. b) Growth of strains whose parent strain is JWCB005 grown in medium containing xylan as the sole source of carbon (reported as whole culture total protein). c) Growth of strains whose parent strain is JWCB018 grown in medium containing xylan as the sole source of carbon (reported as whole culture total protein). d) Growth of strains whose parent strain is JWCB005 grown in medium containing Avicel as the sole source of carbon (reported as whole culture total protein). e) Growth of strains whose parent strain is JWCB018 grown in medium containing Avicel as the sole source of carbon (reported as whole culture total protein). f) Growth of strains whose parent strain is JWCB005 grown in medium containing xylose as the sole source of carbon. g) Growth of strains whose parent strain is JWCB018 grown in medium containing xylose as the sole source of carbon. h) Growth of strains whose parent strain is JWCB005 grown in medium containing glucose as the sole source of carbon. i) Growth of strains whose parent strain is JWCB018 grown in medium containing glucose as the sole source of carbon. j) Growth of strains whose parent strain is JWCB005 grown in medium containing 20 g/L ethanol. k) Growth of strains whose parent strain is JWCB018 grown in medium containing 20 g/L ethanol. l) Growth of strains whose parent strain is JWCB005 grown in medium containing 50 mg/L methyl viologen. m) Growth of strains whose parent strain is JWCB018 grown in medium containing 50 mg/L methyl viologen. n) Growth of strains whose parent strain is JWCB005 grown in medium containing sodium chloride added to a total starting medium osmolarity of 200 mOsm/L. o) Growth of strains whose parent strain is JWCB018 grown in medium containing sodium chloride added to a total starting medium osmolarity of 200 mOsm/L.

**Additional file 2: Figure S2.** PCR of the genomic region spanning B5X54_RS07485–B5X54_RS07500 failed to yield a PCR product when using genomic DNA from strain ORCB002 and primers designed based on the JWCB005 genome, while yielding a product of expected size when using genomic DNA from strain JWCB018 (a derivative of strain JWCB005) as template for PCR. Expected size for PCR product based on JWCB005 genome is 4841 bp. Lane 1 is PCR reaction using ORCB002 genomic DNA as template. Lane 2 is PCR reaction using JWCB018 genomic DNA as template. M1 is O’gene 10 kb ladder (ThermoFisher Scientific catalog # SM1163). M2 is Lambda DNA/HindIII Marker (ThermoFisher Scientific catalog # SM0102).

**Additional file 3: Table S2.** Differential proteomic assessment between strain ORCB012 and its genetic parent strain ORCB002.

**Additional file 4: Figure S3.** Growth profiles and OD_680_ of cultures grown alongside those cultures sampled for RNAseq differential expression analysis of strains ORCB001, ORCB002, and JWCB005 (genetic parent strain to both single-gene deletion strains). OD_680_ values are those of individual culture replicates which were sacrificially sampled and preserved for RNAseq analysis. Growth assays were done in 50 mL culture volumes in 135 mL serum bottles containing a headspace of 100% N_2_.

**Additional file 5: Table S1.** Primers used in this study.

**Additional file 6: Table S3.** Plasmids used in this study.

## Data Availability

Strains generated for this study are available upon request by contacting Miguel Rodriguez Jr. or Dawn Klingeman of the ORNL Biosciences Division. The RNAseq dataset generated and/or analyzed during the current study are available in the NCBI Gene Expression Omnibus (GEO) repository as accession number GSE107393, available at https://www.ncbi.nlm.nih.gov/geo/query/acc.cgi?acc=GSE107393. The dataset generated to assess genomic variations in ORCB002 are available in the NCBI Sequence Read Archive repository as accession number PRJNA543682, available at https://www.ncbi.nlm.nih.gov/sra/?term=PRJNA543682. The proteomic dataset generated to assess heterologous protein expression in ORCB012 are presented in Additional file 3: Table S2 of this published article. All other data generated or analyzed in this article are presented in this published article.
