# Supplementary material for: Gene targets for engineering osmotolerance in Caldicellulosiruptor bescii
Source: Biotechnol Biofuels. 2020 Mar 13;13:50. doi: 10.1186/s13068-020-01690-3 (PMC7071700; doi:10.1186/s13068-020-01690-3)
Supplement: Supplementary file 1 — Additional file 1: Figure S1. Growth or fermentation product analysis of 10 strains considered in this study in various growth conditions relevant to consolidated bioprocessing performance. a) Acetate supernatant concentrations in fermenting cultures of C. bescii grown in medium containing unpretreated switchgrass as the sole source of carbon. b) Growth of strains whose parent strain is JWCB005 grown in medium containing xylan as the sole source of carbon (reported as whole culture total protein). c) Growth of strains whose parent strain is JWCB018 grown in medium containing xylan as the sole source of carbon (reported as whole culture total protein). d) Growth of strains whose parent strain is JWCB005 grown in medium containing Avicel as the sole source of carbon (reported as whole culture total protein). e) Growth of strains whose parent strain is JWCB018 grown in medium containing Avicel as the sole source of carbon (reported as whole culture total protein). f) Growth of strains whose parent strain is JWCB005 grown in medium containing xylose as the sole source of carbon. g) Growth of strains whose parent strain is JWCB018 grown in medium containing xylose as the sole source of carbon. h) Growth of strains whose parent strain is JWCB005 grown in medium containing glucose as the sole source of carbon. i) Growth of strains whose parent strain is JWCB018 grown in medium containing glucose as the sole source of carbon. j) Growth of strains whose parent strain is JWCB005 grown in medium containing 20 g/L ethanol. k) Growth of strains whose parent strain is JWCB018 grown in medium containing 20 g/L ethanol. l) Growth of strains whose parent strain is JWCB005 grown in medium containing 50 mg/L methyl viologen. m) Growth of strains whose parent strain is JWCB018 grown in medium containing 50 mg/L methyl viologen. n) Growth of strains whose parent strain is JWCB005 grown in medium containing sodium chloride added to a total starting medium osmolarity of 200 mOsm/L. o) Gro [file 13068_2020_1690_MOESM1_ESM.docx]

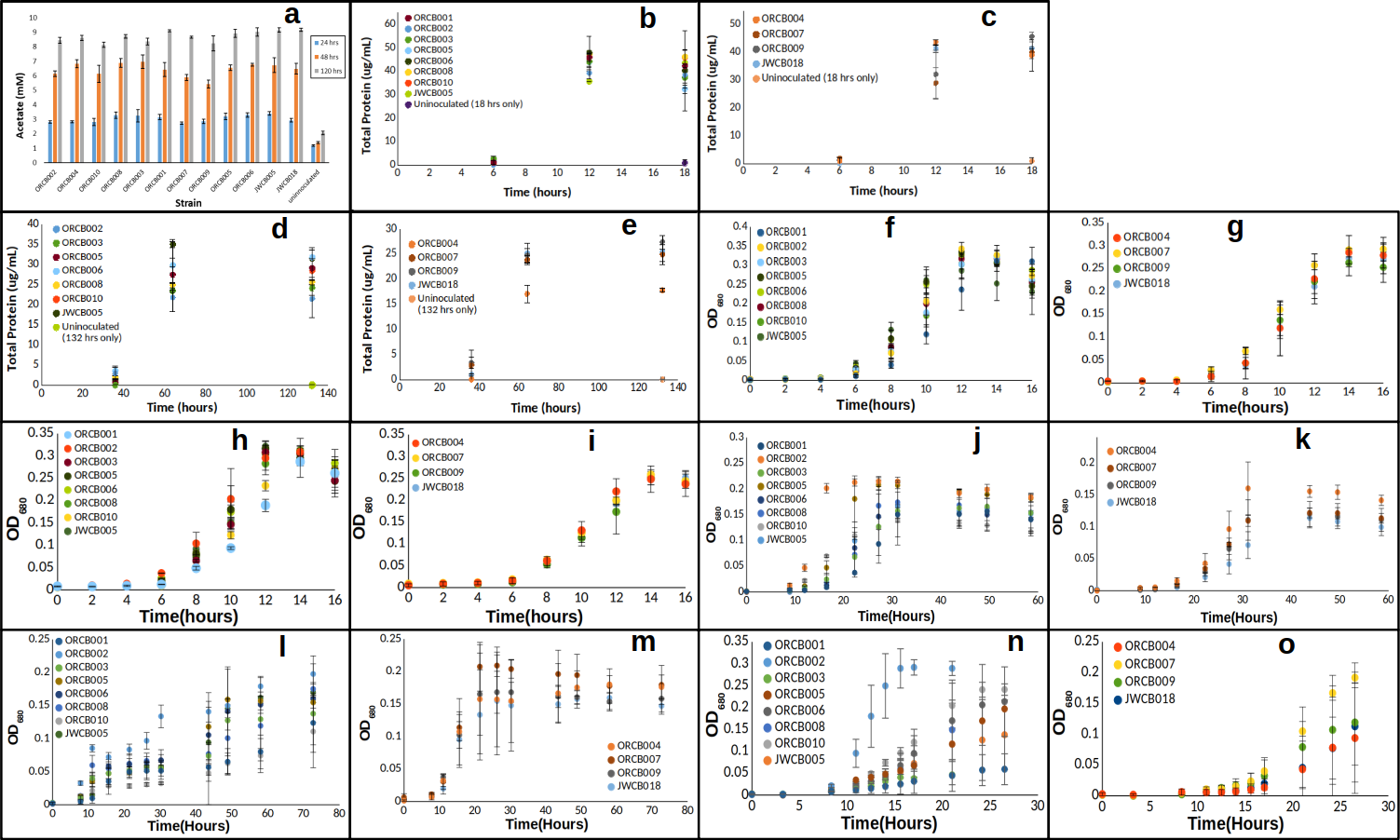


Figure S1: Growth or fermentation product analysis of 10 strains considered in this study in various growth conditions relevant to consolidated bioprocessing performance. a.) Acetate supernatant concentrations in fermenting cultures of *C. bescii* grown in medium containing unpretreated switchgrass as the sole source of carbon b.) Growth of strains whose parent strain is JWCB005 grown in medium containing xylan as the sole source of carbon (reported as whole culture total protein) c.) Growth of strains whose parent strain is JWCB018 grown in medium containing xylan as the sole source of carbon (reported as whole culture total protein) d.) Growth of strains whose parent strain is JWCB005 grown in medium containing avicel as the sole source of carbon (reported as whole culture total protein) e.) Growth of strains whose parent strain is JWCB018 grown in medium containing avicel as the sole source of carbon (reported as whole culture total protein) f.) Growth of strains whose parent strain is JWCB005 grown in medium containing xylose as the sole source of carbon g.) Growth of strains whose parent strain is JWCB018 grown in medium containing xylose as the sole source of carbon h.) Growth of strains whose parent strain is JWCB005 grown in medium containing glucose as the sole source of carbon i.) Growth of strains whose parent strain is JWCB018 grown in medium containing glucose as the sole source of carbon j.) Growth of strains whose parent strain is JWCB005 grown in medium containing 20 g/L ethanol k.) Growth of strains whose parent strain is JWCB018 grown in medium containing 20 g/L ethanol l.) Growth of strains whose parent strain is JWCB005 grown in medium containing 50 mg/L methyl viologen m.) Growth of strains whose parent strain is JWCB018 grown in medium containing 50 mg/L methyl viologen n.) Growth of strains whose parent strain is JWCB005 grown in medium containing sodium chloride added to a total starting medium osmoalrity of 200 mOsm/L o.) Growth of strains whose parent strain is JWCB018 grown in medium containing sodium chloride added to a total starting medium osmoalrity of 200 mOsm/L
