# Supplementary material for: Gene targets for engineering osmotolerance in Caldicellulosiruptor bescii
Source: Biotechnol Biofuels. 2020 Mar 13;13:50. doi: 10.1186/s13068-020-01690-3 (PMC7071700; doi:10.1186/s13068-020-01690-3)
Supplement: Supplementary file 2 — Additional file 2: Figure S2. PCR of the genomic region spanning B5X54_RS07485–B5X54_RS07500 failed to yield a PCR product when using genomic DNA from strain ORCB002 and primers designed based on the JWCB005 genome, while yielding a product of expected size when using genomic DNA from strain JWCB018 (a derivative of strain JWCB005) as template for PCR. Expected size for PCR product based on JWCB005 genome is 4841 bp. Lane 1 is PCR reaction using ORCB002 genomic DNA as template. Lane 2 is PCR reaction using JWCB018 genomic DNA as template. M1 is O’gene 10 kb ladder (ThermoFisher Scientific catalog # SM1163). M2 is Lambda DNA/HindIII Marker (ThermoFisher Scientific catalog # SM0102). [file 13068_2020_1690_MOESM2_ESM.docx]

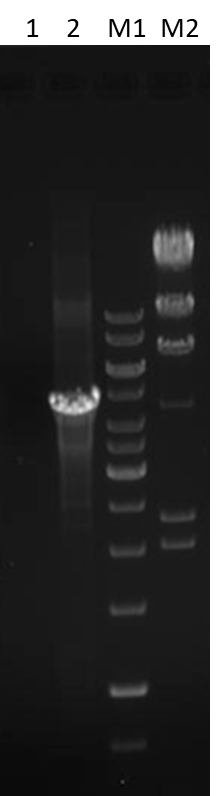


Figure S2. PCR of the genomic region spanning B5X54_RS07485 - B5X54_RS07500 failed to yield a PCR product when using genomic DNA from strain ORCB002 and primers designed based on the JWCB005 genome, while yielding a product of expected size when using genomic DNA from strain JWCB018 (a derivative of strain JWCB005) as template for PCR. Expected size for PCR product based on JWCB005 genome is 4841 bp. Lane 1 is PCR reaction using ORCB002 genomic DNA as template. Lane 2 is PCR reaction using JWCB018 genomic DNA as template. M1 is O’gene 10 kb ladder (ThermoFisher Scientific catalog # SM1163). M2 is Lambda DNA/HindIII Marker (ThermoFisher Scientific catalog # SM0102).
