# Supplementary material for: Gene targets for engineering osmotolerance in Caldicellulosiruptor bescii
Source: Biotechnol Biofuels. 2020 Mar 13;13:50. doi: 10.1186/s13068-020-01690-3 (PMC7071700; doi:10.1186/s13068-020-01690-3)
Supplement: Supplementary file 4 — Additional file 4: Figure S3. Growth profiles and OD680 of cultures grown alongside those cultures sampled for RNAseq differential expression analysis of strains ORCB001, ORCB002, and JWCB005 (genetic parent strain to both single-gene deletion strains). OD680 values are those of individual culture replicates which were sacrificially sampled and preserved for RNAseq analysis. Growth assays were done in 50 mL culture volumes in 135 mL serum bottles containing a headspace of 100% N2. [file 13068_2020_1690_MOESM4_ESM.docx]

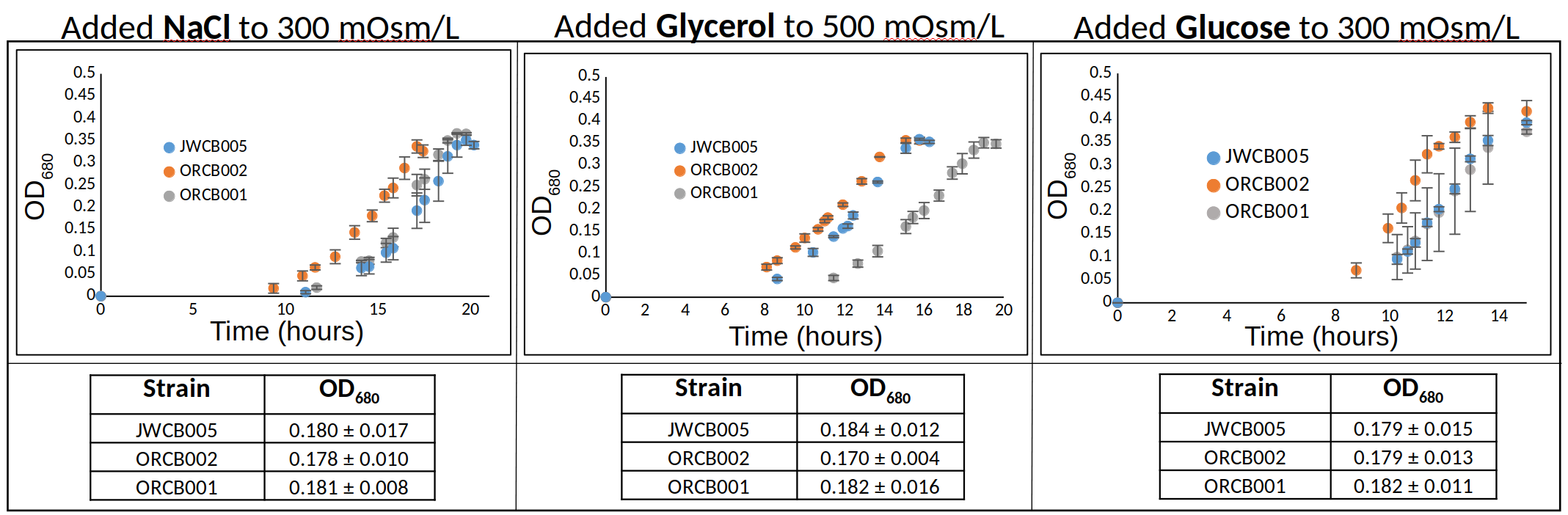


Figure S3. Growth profiles and OD_680_ of cultures grown alongside those cultures sampled for RNAseq differential expression analysis of strains ORCB001, ORCB002, and JWCB005 (genetic parent strain to both single-gene deletion strains). OD_680_ values are those of individual culture replicates which were sacrificially sampled and preserved for RNAseq analysis. Growth assays were done in 50 mL culture volumes in 135 mL serum bottles containing a headspace of 100% N_2_.
