# Supplementary material for: Gene targets for engineering osmotolerance in Caldicellulosiruptor bescii
Source: Biotechnol Biofuels. 2020 Mar 13;13:50. doi: 10.1186/s13068-020-01690-3 (PMC7071700; doi:10.1186/s13068-020-01690-3)
Supplement: Supplementary file 5 — Additional file 5: Table S1. Primers used in this study. [file 13068_2020_1690_MOESM5_ESM.docx]

Table S1. Primers used in this study

| Primer Name | Primer Sequence | Purpose |
| --- | --- | --- |
| upstm 5' flank fwd athe_1015_P3 | TGGGCTACTCCTTTGAGGAA | genomic screening primer flanking the B5X54_RS05670 gene |
| dnstm 3' flank rev athe_1015_P3 | CAATTGAATTTCCCGACCTG | genomic screening primer flanking the B5X54_RS05670 gene |
| internal rev athe_1015_P3 | GCTCCTGGTTGAAACCAAAC | genomic screening primer binding internal to the B5X54_RS05670 gene |
| upstm 5' flank fwd athe_1669_P3 | GGGATATCGCTATAAGATGAAGG | genomic screening primer flanking the B5X54_RS08965 gene |
| dnstm 3' flank rev athe_1669_P3 | TTTCGGAATATAATTGTGCATGA | genomic screening primer flanking the B5X54_RS08965 gene |
| internal rev athe_1669_P3 | TTCACGAAAAGAAGGAGACCA | genomic screening primer binding internal to the B5X54_RS08965 gene |
| upstm 5' flank fwd athe_1152_P3 | CGTCAAAAAGAGCCAAGAGG | genomic screening primer flanking the B5X54_RS06355 gene |
| dnstm 3' flank rev athe_1152_P3 | TCAGGGGGTGAAAATGGTAA | genomic screening primer flanking the B5X54_RS06355 gene |
| internal rev athe_1152_P3 | AAATTTGCCCCTGAAAACCT | genomic screening primer binding internal to the B5X54_RS06355 gene |
| upstm 5' flank fwd athe_2264_P3 | GCATAACTCCTAATGAATGTCCAA | genomic screening primer flanking the B5X54_RS12050 gene |
| dnstm 3' flank rev athe_2264_P3 | GAGGTGCTGCGTATGTTCTG | genomic screening primer flanking the B5X54_RS12050 gene |
| internal rev athe_2264_P3 | GCTGCAGCAGACCTGTACG | genomic screening primer binding internal to the B5X54_RS12050 gene |
| upstm 5' flank fwd athe_0146_P3 | ACCTGCCACCACAACTACAA | genomic screening primer flanking the B5X54_RS01260 gene |
| dnstm 3' flank rev athe_0146_P3 | GCACCCTTTGATCCCATCG | genomic screening primer ackflanking the B5X54_RS01260 gene |
| internal rev athe_0146_P3 | CAAGCCTTTCCACAGGACAT | genomic screening primer binding internal to the B5X54_RS01260 gene |
| upstm 5' flank fwd athe_0343_P3 | GAGAGTTCTGTGGGGCAAAA | genomic screening primer flanking the B5X54_RS02215 gene |
| dnstm 3' flank rev athe_0343_P3 | ATCTTGTGAACGCCAAGGTC | genomic screening primer flanking the B5X54_RS02215 gene |
| internal rev athe_0343_P3 | GGCTGTGCAACTGTTGTGAT | genomic screening primer binding internal to the B5X54_RS02215 gene |
| upstm 5' flank fwd athe_0799_P3 | CGGTGATGTAACCACTCCAA | genomic screening primer flanking the B5X54_RS04585 gene |
| dnstm 3' flank rev athe_0799_P3 | TTCCAATACCGCTTTTAAATGCCT | genomic screening primer flanking the B5X54_RS04585 gene |
| internal rev athe_0799_P3 | TAATCTTGACTGCCGTGCATC | genomic screening primer binding internal to the B5X54_RS04585 gene |
| upstm 5' flank fwd athe_2074_P3 | CACCCACTGTATTGCCCTTT | genomic screening primer flanking the B5X54_RS11065 gene |
| dnstm 3' flank rev athe_2074_P3 | AACACATGGGAGGAGACCAG | genomic screening primer flanking the B5X54_RS11065 gene |
| internal rev athe_2074_P3 | GAAGTGAATGAGATTGCAAGAGGC | genomic screening primer binding internal to the B5X54_RS11065 gene |
| upstm 5' flank fwd athe_1407_P3 | CTGCTGCTCTTGCTCTCCTT | genomic screening primer flanking the B5X54_RS07610 gene |
| dnstm 3' flank rev athe_1407_P3 | CAAAGTTGGAACAATGATTGAGG | genomic screening primer flanking the B5X54_RS07610 gene |
| internal rev athe_1407_P3 | GGGCAGGAAGTTGAAAAACA | genomic screening primer binding internal to the B5X54_RS07610 gene |
| pyr fwd screen | CTTGAAAATCCAGGGGTTGA | pyrF region to screen pyr biosyntheis region of gDNA of delAthe_0146 mutant |
| pyr rev screen | GGTCAGTTTTCCCTTGGACA | pyrF region to screen pyr biosyntheis region of gDNA of delAthe_0146 mutant |
| pJGW03 fwd insert check seq | gccgcatctgagagtt | screening insert site on pJGW03-based integrating suicide vectors |
| pJGW03 rev insert check seq | ctacggaaggagctgtg | screening insert site on pJGW03-based integrating suicide vectors |
| pDCW88 gib assy backbone fwd | gtgcactctgacgctc | amplify pJGW03 based integrating suicide vector backbone |
| pDCW88 gib assy backbone rev | ggtaccaccagcctaac | amplify pJGW03 based integrating suicide vector backbone |
| pDCW88_athe_1152_up_fwd | tccaatgatcgaagttaggctggtggtaccAATCTATAAGGTAAAGGACCTTGTTG | amplify upstream region of the B5X54_RS06355 gene |
| pDCW88_athe_1152_up_rev | AATCCTCATATTTTACTACACTCCCATTTCAATTTTATCACCATGATTTAATATTTGA | amplify upstream region of the B5X54_RS06355 gene |
| pDCW88_athe_1152_down_fwd | GAAATGGGAGTGTAGTAAAATATGAG | amplify downstream region of the B5X54_RS06355 gene |
| pDCW88_athe_1152_down_rev | gttttcgttccactgagcgtcagagtgcacgccatctgagcaaactgc | amplify downstream region of the B5X54_RS06355 gene |
| pDCW88_athe_1015_up_fwd | tccaatgatcgaagttaggctggtggtaccATAAAATTTAAAGAGGCAGCTTTAGC | amplify upstream region of the B5X54_RS05670 gene |
| pDCW88_athe_1015_up_rev | AAGCGCAGATGGCGGTAAATTTTTGAAAAGatctttccacctcattttctg | amplify upstream region of the B5X54_RS05670 gene |
| pDCW88_athe_1015_down_fwd | CTTTTCAAAAATTTACCGCC | amplify downstream region of the B5X54_RS05670 gene |
| pDCW88_athe_1015_down_rev | gttttcgttccactgagcgtcagagtgcacAATACCAGAGCCAACACCTG | amplify downstream region of the B5X54_RS05670 gene |
| pDCW88_athe_0343_up_fwd | tccaatgatcgaagttaggctggtggtaccAAATTTAAAAGAACAAAAACATGATACTG | amplify upstream region of the B5X54_RS02215 gene |
| pDCW88_athe_0343_up_rev | acaatggttttaaaataaaaaataaccttcTTTACACCCCCAGATACTTTTAC | amplify upstream region of the B5X54_RS02215 gene |
| pDCW88_athe_0343_down_fwd | GAAGGTTATTTTTTATTTTAAAACCATTG | amplify downstream region of the B5X54_RS02215 gene |
| pDCW88_athe_0343_down_rev | gttttcgttccactgagcgtcagagtgcacGCCTTTTTGTGGGCTATATTTAAC | amplify downstream region of the B5X54_RS02215 gene |
| pDCW88_athe_2074_up_fwd | tgatcgaagttaggctggtggtaccGAAAGATAAGACTTATTTTTCATTATAGAAACTG | amplify upstream region of the B5X54_RS11065 gene |
| pDCW88_athe_2074_up_rev | aactaataaaaggttattatacaaaagaggTTTTATTGGGTTTGAGATGATTTG | amplify upstream region of the B5X54_RS11065 gene |
| pDCW88_athe_2074_down_fwd | CCTCTTTTGTATAATAACCTTTTATTAGTTTA | amplify downstream region of the B5X54_RS11065 gene |
| pDCW88_athe_2074_down_rev | gttttcgttccactgagcgtcagagtgcacGGATTTAGTTCTACACCCAAAATAG | amplify downstream region of the B5X54_RS11065 gene |
| pDCW88_athe_2264_up_fwd | ccaatgatcgaagttaggctggtggtaccAAACTCATATAATTTTTCACTCATTTTTTC | amplify upstream region of the B5X54_RS12050 gene |
| pDCW88_athe_2264_up_rev | AAAATTATATTTGAGGAGGTTTGGTAGGCTatgaactgagatgtatcttaaaaagc | amplify upstream region of the B5X54_RS12050 gene |
| pDCW88_athe_2264_down_fwd | AGCCTACCAAACCTCCTCA | amplify downstream region of the B5X54_RS12050 gene |
| pDCW88_athe_2264_down_rev | gttttcgttccactgagcgtcagagtgcacTGTGTACTATGTTGGTAGTAACGAAA | amplify downstream region of the B5X54_RS12050 gene |
| pDCW88_athe_1407_up_fwd | tccaatgatcgaagttaggctggtggtaccCTCCTCATTTACACCCATAACA | amplify upstream region of the B5X54_RS07610 gene |
| pDCW88_athe_1407_up_rev | TAAAATTGTCCCGCTGGAGAATGAGAGAGTaaggtaagaggcaaaaacactg | amplify upstream region of the B5X54_RS07610 gene |
| pDCW88_athe_1407_down_fwd | ACTCTCTCATTCTCCAGCG | amplify downstream region of the B5X54_RS07610 gene |
| pDCW88_athe_1407_down_rev | gttttcgttccactgagcgtcagagtgcacATGACTTAACACAGATGACTTTTGG | amplify downstream region of the B5X54_RS07610 gene |
| pDCW88_athe_0799_up_fwd | tccaatgatcgaagttaggctggtggtaccAATTGGAGAAAAACTGTCTTCAAAG | amplify upstream region of the B5X54_RS04585 gene |
| pDCW88_athe_0799_up_rev | cccttgaccatttttcattactcctcttttCTTATCCATCTATCTTCATCTCTACCG | amplify upstream region of the B5X54_RS04585 gene |
| pDCW88_athe_0799_down_fwd | AAAAGAGGAGTAATGAAAAATGGTC | amplify downstream region of the B5X54_RS04585 gene |
| pDCW88_athe_0799_down_rev | gttttcgttccactgagcgtcagagtgcacCTTCATATCTGCCACTTTCAAG | amplify downstream region of the B5X54_RS04585 gene |
| pDCW88_athe_1669_up_fwd | tccaatgatcgaagttaggctggtggtaccTTGTTCTAATGTAAGATCTGAAAACAATC | amplify upstream region of the B5X54_RS08965 gene |
| pDCW88_athe_1669_up_rev | tcagatgagcagaaatgaggtatataaaagGATGGTTAAAGATGAGATTGTAAAGG | amplify upstream region of the B5X54_RS08965 gene |
| pDCW88_athe_1669_down_fwd | CTTTTATATACCTCATTTCTGCTCATC | amplify downstream region of the B5X54_RS08965 gene |
| pDCW88_athe_1669_down_rev | tttcgttccactgagcgtcagagtgcacTATAAAGAGTTAAAAAGAGGAGATTAAAGAT | amplify downstream region of the B5X54_RS08965 gene |
| pDCW88_athe_0146_up_fwd | tccaatgatcgaagttaggctggtggtaccCGACTTTGTATACAACCCATCTTC | amplify upstream region of the B5X54_RS01260 gene |
| pDCW88_athe_0146_up_rev | caatttgatcccaccttgaaatttaaaattTTTATTCACCTTTCGTTTATGC | amplify upstream region of the B5X54_RS01260 gene |
| pDCW88_athe_0146_down_fwd | AATTTTAAATTTCAAGGTGGGATC | amplify downstream region of the B5X54_RS01260 gene |
| pDCW88_athe_0146_down_rev | gttttcgttccactgagcgtcagagtgcacTTTCGTTTTAGCATTCTTATAAGCTC | amplify downstream region of the B5X54_RS01260 gene |
| fwd amp_pDCW173::0146_overlap | GAGTATGAAAAACTTAGTGTGAAGGTCATCACGTAAgagggtgagattgattctcac | amplifying replicating shuttle vector backbone containing B5X54_RS01260 gene |
| rev amp_pDCW173::0146_overlap | CTGCGCAATTCTGCTTTTTCTTTCCTCTGCAAACATaactactcaccaaacctccttg | amplifying replicating shuttle vector backbone containing B5X54_RS01260 gene |
| fwd athe_0146::pDCW173 insert | aaatcatacaaggaggtttggtgagtagttATGTTTGCAGAGGAAAGAAAA | amplifying B5X54_RS01260 gene for expression insert |
| rev athe_0146::pDCW173 insert | taaaagagggtgagaatcaatctcaccctcTTACGTGATGACCTTCACACTA | amplifying B5X54_RS01260 gene for expression insert |
| fwd amp_pDCW173::1152_overlap | GTTGCATTAAATGAAGAGACTAATTCAGCGCAATAGgagggtgagattgattctcac | amplifying replicating shuttle vector backbone containing B5X54_RS06355 gene |
| rev amp_pDCW173::1152_overlap | AAGTCTGTGTCTTTCTTTCCTTGATAGTTTTGCCATaactactcaccaaacctccttg | amplifying replicating shuttle vector backbone containing B5X54_RS06355 gene |
| fwd athe_1152::pDCW173 insert | aaatcatacaaggaggtttggtgagtagttATGGCAAAACTATCAAGGAAAG | amplifying B5X54_RS06355 gene for expression insert |
| rev athe_1152::pDCW173 insert | taaaagagggtgagaatcaatctcaccctcCTATTGCGCTGAATTAGTCTCTT | amplifying B5X54_RS06355 gene for expression insert |
| Forward (Gblk_fwd) - Kan HT | ATGAAAGGACCTATAATTATG | amplifying high-temperature kanamycin marker for automously replicating expression vectors |
| Reverse (Gblk_rev) - Kan HT | TCAAAATGGTATTCTTTTG | amplifying high-temperature kanamycin marker for automously replicating expression vectors |
| fwd_kaninsert_07_XbaI | AACTTTCTACATAGAAAGGATGGTCTCTAGatgaaaggacctataattatgactagag | amplifying high-temperature kanamycin marker for automously replicating expression vectors |
| rev_kaninsert_07_NdeI | CGTTACATATCAAAGGGAAAACTGTCCATAtcaaaatggtattcttttgctaacatc | amplifying high-temperature kanamycin marker for automously replicating expression vectors |
| fwd_mkrscreen_pJGW07 | atccgttgatcttcctgcat | screening marker site on autonomously replicating expression vectors |
| rev_mkrscreen_pJGW07 | ctcacgcaaaaacaacgaac | screening marker site on autonomously replicating expression vectors |
| fwd amp173_Mkr::Kan overlap | AGTAGATGTTAGCAAAAGAATACCATTTTGAcatcatcatcatcatcactaacc | amplifying expression vector backbones to insert high-temperature kanamycin marker |
| rev amp173_Mkr::Kan overlap | TCTTCTCTAGTCATAATTATAGGTCCTTTCATtctagagaccatcctttctatg | amplifying expression vector backbones to insert high-temperature kanamycin marker |
