# Supplementary material for: Gene targets for engineering osmotolerance in Caldicellulosiruptor bescii
Source: Biotechnol Biofuels. 2020 Mar 13;13:50. doi: 10.1186/s13068-020-01690-3 (PMC7071700; doi:10.1186/s13068-020-01690-3)
Supplement: Supplementary file 6 — Additional file 6: Table S3. Plasmids used in this study. [file 13068_2020_1690_MOESM6_ESM.docx]

Table S3. Plasmids used in this study

| Plasmid | Purpose |
| --- | --- |
| pB5X54_RS06355_del | Non-replicating vector used to delete the B5X54_RS06355 gene |
| pB5X54_RS05670_del | Non-replicating vector used to delete the B5X54_RS05670 gene |
| pB5X54_RS02215_del | Non-replicating vector used to delete the B5X54_RS02215 gene |
| pB5X54_RS11065_del | Non-replicating vector used to delete the B5X54_RS11065 gene |
| pB5X54_RS12050_del | Non-replicating vector used to delete the B5X54_RS12050 gene |
| pB5X54_RS07610_del | Non-replicating vector used to delete the B5X54_RS07610 gene |
| pB5X54_RS04585_del | Non-replicating vector used to delete the B5X54_RS04585 gene |
| pB5X54_RS08965_del | Non-replicating vector used to delete the B5X54_RS08965 gene |
| pB5X54_RS01260_del | Non-replicating vector used to delete the B5X54_RS01260 gene |
| pJGW07::Kan | Autonomously replicating empty vector |
| pJGW07::B5X54_RS01260::Kan | Autonomously replicating vector expressing the B5X54_RS01260 gene |
| pJGW07::B5X54_RS06355::Kan | Autonomously replicating vector expressing the B5X54_RS06355 gene |
